# The Impact of Hepatic Artery Thrombosis on the Outcome of Pediatric Living Donor Liver Transplantations

**DOI:** 10.3390/children10020340

**Published:** 2023-02-09

**Authors:** Marek Stefanowicz, Piotr Kaliciński, Grzegorz Kowalewski, Adam Kowalski, Mateusz Ciopiński, Marek Szymczak, Agnieszka Kwiecińska, Waldemar Patkowski, Krzysztof Zieniewicz, Ireneusz Grzelak, Diana Kamińska, Hor Ismail

**Affiliations:** 1 Department of Pediatric Surgery and Organ Transplantation, The Children’s Memorial Health Institute, 04-730 Warsaw, Poland; 2 Department of General Surgery, Transplantation and Liver Surgery, Warsaw Medical University, 02-091 Warsaw, Poland; 3 Department of Gastroenterology, Hepatology, Nutritional Disorders and Pediatrics, The Children’s Memorial Health Institute, 04-730 Warsaw, Poland

**Keywords:** hepatic artery thrombosis, liver transplantation, living donor

## Abstract

The aim of our study was to assess risk factors for hepatic artery thrombosis (HAT) and to evaluate the impact of HAT management on long-term outcomes after pediatric living donor liver transplantation (LDLT). We retrospectively analyzed 400 patients who underwent primary LDLT between 1999 and 2020. We compared preoperative data, surgical factors, complications, and patient and graft survivals in patients with HAT (HAT Group) and without HAT (non-HAT Group). A total of 27 patients (6.75%) developed HAT. Acute liver failure, a hepatic artery (HA) anastomosis diameter below 2 mm, and intraoperative HA flow dysfunction were significantly more common in the HAT Group (*p* < 0.05, *p* = 0.02026, and *p* = 0.0019, respectively). In the HAT Group, 21 patients (77.8%) underwent urgent surgical revision. The incidence of biliary stenosis and retransplantation was significantly higher in the HAT Group (*p* = 0.00002 and *p* < 0.0001, respectively). Patient and graft survivals were significantly worse in the HAT Group (*p* < 0.05). The close monitoring of HA flow with Doppler ultrasound during the critical period of 2 to 3 weeks after LDLT and the immediate attempt of surgical revascularization may attenuate the elevated risk of biliary stenosis, graft loss, and the need for retransplantation due to HAT.

## 1. Introduction

The reconstruction of the hepatic artery (HA) is the most challenging vascular anastomosis in pediatric living donor liver transplantation (LDLT). Both the small size and the number of anatomic variations on the donor and recipient sides make this anastomosis extremely difficult to perform. The patency of the anastomosis determines the outcome of both the graft and the recipient. The development of hepatic artery thrombosis (HAT) after pediatric LDLT is a severe complication associated with high morbidity and mortality, as HAT can lead to graft necrosis, abscess, and biliary complications (due to ischemia of the biliary tract). 

There are two types of HAT. Early HAT (eHAT) occurs within the first 30 days post LDLT. Arterial thrombosis after 30 days is classified as late HAT. The incidence of HAT in pediatric LDLT varies between 1% and 26% [1]. Early HAT is a major cause of graft loss and mortality in the postoperative period after liver transplantation [2]. It remains one of the main indications for retransplantation [3]. 

Early diagnosis and intervention may alleviate these complications and improve graft and patient survival. The best results are obtained with immediate arterial revascularization, otherwise, retransplantation is necessary.

The aim of our study was to assess potential risk factors for HAT and to evaluate the impact of HAT management on long-term outcomes after LDLT in pediatric patients.

## 2. Materials and Methods

We have retrospectively reviewed a cohort of 400 patients after primary LDLT performed in our hospital between 12 October 1999 and 31 December 2020. Patients after retransplantation with a living donor graft were excluded from the study. Demographic and clinical details from donor and recipient hospital records, as well as data from the National Transplant Registry, were collected. Surgical information about each patient came from the surgical reports. To evaluate the relationship between HAT and recipient outcome, we compared preoperative data, surgical factors, complications, and survival between patients with HAT (HAT group) and without HAT (non-HAT group). Diagnostic and therapeutic interventions for HAT were also analyzed.

The analysis compared variables related to the recipient, donor, and surgery. Pre-transplant variables included underlying liver disease, recipient and donor ages, gender, weight at time of transplantation, pediatric end-stage liver disease (PELD) score, and donor–recipient ABO matching. Intra-operative parameters, such as graft weight, graft-to-recipient weight ratio (GRWR), liver graft type, biliary anastomosis type, cold ischemia time (CIT), duration of vascular anastomosis, duration of hepatic artery anastomosis, number of HA anastomoses, diameter of anastomosis, performance of intraoperative hepatic artery revision, and need for delayed abdominal wound closure, were also analyzed. Post-transplant outcomes included HAT development, biliary leakage from biliary anastomosis, biliary stricture, retransplantation, and death. Biliary leakage was diagnosed when bile-like ascites occurred in a drain or in the presence of biloma. A biliary stricture was defined as stenosis of a biliary anastomosis and the need for percutaneous drainage and/or dilatation, or surgical revision of the anastomosis. Graft and patient survival were evaluated and compared between the study groups.

### 2.1. Donors

Liver grafts were harvested from healthy adult donors who gave informed consent. The graft from a living donor was procured in an adult transplantation surgery unit with significant experience in hepatobiliary surgery. In all cases, liver grafts were perfused with UW solution through the portal vein and the hepatic artery immediately after retrieval.

### 2.2. Recipient Surgery and Hepatic Artery Anastomosis

Recipient surgery was done using a standard technique. The recipient’s right and left hepatic arteries were preserved with maximum length and were trimmed before HA anastomosis, according to the length of the donor artery. During hepatectomy the native liver was removed and the retrohepatic inferior vena cava was left. To obtain adequate blood outflow, a wide ostium from the middle and left hepatic vein was created, and the vein of the liver graft was sutured in triangular fashion with three continuous sutures. The portal vein (PV) end-to-end anastomosis was performed with two running 6/0 or 7/0 monofilament resorbable sutures.

Hepatic artery anastomosis was performed as the last vessel under 3.5× magnification with surgical loupes. To avoid vascular injury and prevent intimal damage, the no touch technique was used, and a vascular clamp was applied only on the recipient side of the HA.

In the case of multiple arteries, backflow was always checked at the back-table preparation. If possible, multiple arterial anastomoses were performed depending on the anatomy of the graft and the recipient’s arteries. Larger HAs were used for reconstruction first. If back flow was not visualized, double arterial anastomosis, or another reconstruction, was mandatory to provide arterial inflow to the whole graft.

**Figure 1:**
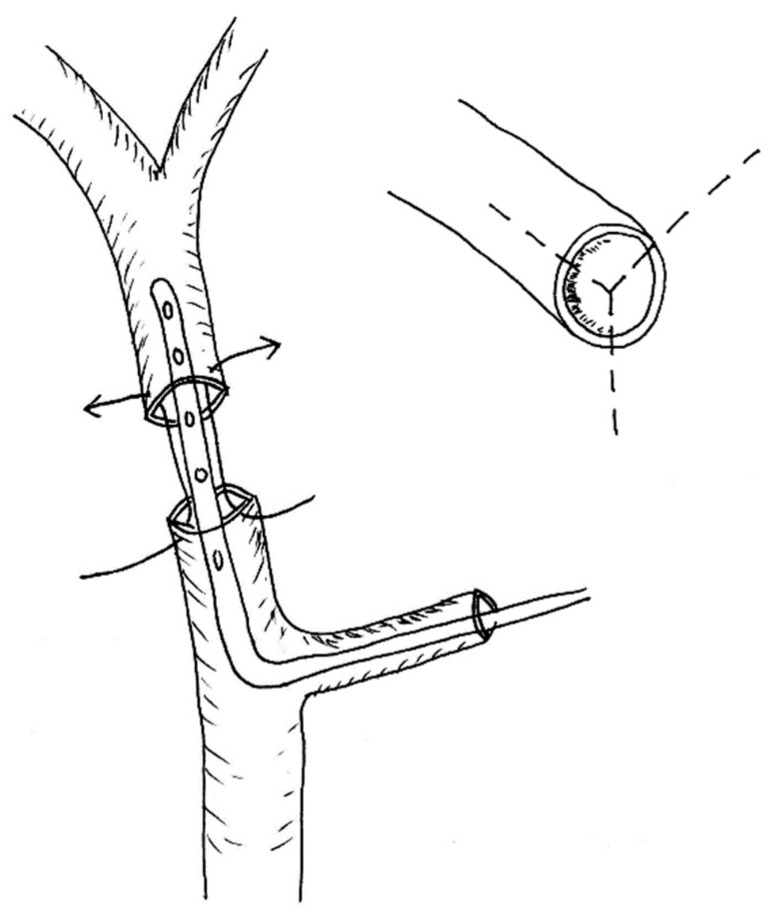
End-to-end HA anastomosis with three angel sutures.

The end-to-end hepatic artery anastomoses were performed with non-absorbable 8/0 interrupted sutures. Whenever possible, perfusion via a microcatheter (4F) introduced across the anastomosis (normally through the gastroduodenal artery or branch of HA) was applied to keep the vessels open during suturing (Figure 1). We usually began the anastomosis with three angle sutures to keep it open and to facilitate the placement of the remaining sutures.

The vascular anastomosis duration was calculated from the start of hepatic vein suturing to blood flow establishment. The duration of HA anastomosis was calculated from the start to the end of arterial suturing. 

Cold ischemia time was defined as the time between the start of the cold perfusion of the graft and removal of the liver from ice and the start of vascular anastomosis. When performing vascular anastomoses, however, the graft was flushed continuously through the portal vein with a cold 2.5% albumin solution to avoid warm ischemia. Graft blood flow was restored after completing all vascular anastomoses.

Intraoperative color Doppler ultrasonography was performed routinely after graft reperfusion and at the end of surgery. The blood flow through all the anastomoses was evaluated. The immediate revision of HA or PV anastomosis was done, usually with the introduction of a Fogarty catheter, when no flow was found in the HA or PV, minimal portal flow was detected with absence of flow in part of the lumen, or when the HA resistive index RI = 1 with the complete absence of the diastolic signal and very low systolic velocity. In case of anastomotic kinking or narrowing, reanastomosis had to be performed.

For bile duct reconstruction, either the Roux-en-Y hepaticojejunostomy or, less commonly, duct-to-duct anastomosis were performed. Abdominal closure was delayed in case of wound tension and possible graft compression, with the temporary (3 to 5 days) implantation of prosthetic material (Vicryl mesh) at the surgeon’s discretion.

### 2.3. Postoperative Monitoring and Management

A Doppler ultrasound was routinely performed twice a day during the first 5–7 days after liver transplantation, then once a day for the next week, and at every check-up. It was also performed when clinically indicated (e.g., coagulopathy, elevated liver enzymes, fever) or before/after surgical reoperations due to other reasons (e.g., bleeding). 

The routine postoperative anticoagulation regimen consisted of enoxaparin at a dose of 0.5 to 0.75 mg/kg twice a day administered for 2 to 3 weeks, with a transition to acetylsalicylic acid at a dose of 1 mg/kg once a day for six months. After surgery, the central venous pressure was maintained at around 4 to 6 cm H_2_O, and the blood pressure was kept at upper normal limits with the support of dopamine and noradrenaline, if necessary. Hematocrit was kept at about 25% to 28%, and blood transfusions were not given without a surgeon’s decision. FFP was administered only if INR > 3.

Primary immunosuppression consisted of tacrolimus and mycophenolate mofetil. Steroids were given only when indicated, with individual modifications.

### 2.4. HAT Diagnosis and Revascularization Technique

HAT was defined as the absence of intrahepatic arterial flow in ultrasound examination. In patients with two arterial anastomoses, HAT was also diagnosed when intrahepatic arterial flow was absent. If flow was present, even via only one HA, we did not consider it HAT. Computed tomography (CT) to confirm HAT was not routinely performed. 

The decision to surgically intervene was made based on the time from transplant surgery, graft function, and the assessment of the anatomical possibility of HA reconstruction. Surgical revascularization was preferred over conservative treatment. Surgery was performed immediately after the diagnosis of HAT. 

During the surgery, HAT was confirmed by the examination of the hepatic artery with no intrahepatic arterial flow in Doppler ultrasound. Thrombectomy with a Fogarty catheter was performed either through a branch of the hepatic artery or the anastomosis between the donor and recipient’s arteries. Subsequently, the hepatic artery was flushed with a combination of saline and heparin. If back bleeding from the graft artery was not obtained, a catheter was inserted into the hepatic artery, and the intra-arterial infusion of recombinant activated tissue plasminogen (r-TPA) at a dose of 0.05 mg/kg for 20 to 30 min was performed. In the case of stenosis, kinking, or angulation of the HA anastomosis, new arterial anastomoses were created after shortening the donor and recipient hepatic arteries. Reanastomosis was performed with 8/0 monofilament interrupted sutures. 

A successful revascularization attempt was defined as the presence of normal blood flow via the hepatic artery in Doppler ultrasound at follow-up examinations up to 12 months after transplantation. 

### 2.5. Statistical Analysis

To analyze our data, we used Statistica 13 software, Statsoft inc (Tulsa, USA). We created Kaplan–Meier curves to estimate survival and used log-rank or Cox-Mantel tests to assess differences between groups. Baseline demographics and clinical data were presented as medians, ranges, and distributions for categorical variables. Unpaired associations between continuous variables were evaluated using the Student t-test and Mann–Whitney U test. We compared categorical variables using the Chi-Square test of independence. A p-value of less than 0.05 was considered statistically significant. 

Our study received the approval of the institutional Ethics Committee (approval number: 13/KBE/2022).

## 3. Results

### 3.1. Recipients, Donors, and Grafts

Between October 1999 and December 2020, at our institution, we performed 406 LDLT in 402 patients younger than 18 years old. We included 400 patients who received primary LDLT in our study. Six cases of retransplantation were excluded from the study: two patients who received their first liver transplantation from a deceased donor and four who underwent retransplantation from a living donor after previous LDLT. The follow-up time ranged from one day to 22 years, a median of eight years (ranging from one day to 22 years).

Of the 400 children, 27 (6.75%) developed HAT (HAT group). In 25 children (92.6%), HAT was diagnosed within 30 days after LDLT and, in two cases, after more than 30 days. The median time from LDLT to a diagnosis of HAT was six days (ranging from 2.5 hours to 116 days). 

The most common primary liver disease in the HAT group was cholestatic disease in 16 patients (59.3%), including 14 patients with biliary atresia, followed by acute liver failure in five children (18.5% ) and liver tumors in two patients (7.4%) (Table 1). The indications for liver replacement in the non-HAT group were cholestatic liver disease in 248 patients (66.5%), including 222 with biliary atresia, liver tumors in 47 (12.6%), and acute liver failure in 28 (7.5%). Before LDLT, we performed Kasai hepatoportoenterostomy in all patients with biliary atresia. The number of children with acute liver failure was significantly higher in the HAT group (*p* < 0.05).

There were no significant differences in patient characteristics between the groups (Table 2). There were 16 female (59.3%) recipients in the HAT group. Their median age, body weight, and PELD score at the time of transplantation were 1.3 years, 9.5 kg, and 12.5, respectively. In the non-HAT group, there were 197 females (52.8%) with a median age, body weight, and PELD score at the time of transplantation of 1.2 years, 9.5 kg, and 14, respectively. The number of patients who underwent AB0-incompatible liver transplantation and the number of urgent LDLT did not differ between the groups (*p* = 0.92 and *p* = 0.2, respectively). The analyses revealed no differences in donor sex, age, and body weight at the time of LDLT between the two groups.

Various surgical factors are presented in Table 3. In the HAT group, 24 patients received a left lateral segment, whereas three received a left lobe. In the non-HAT group, the grafts consisted of left lateral segments in 321 patients and the left lobe in 38 patients. Thirteen children without HAT received a monosegmental graft, and one of them received the right lobe. The graft weight and median graft-to-recipient weight ratio (GRWR) were 256 g and 2.2% in the HAT group and 268 g and 2.7% in the non-HAT group, respectively. No significant differences between groups were found in graft weight and GRWR (*p* = 0.78 and 0.19, respectively). 

Vascular anastomosis, HA anastomosis, and cold ischemia times were 60.5 min, 17 min, and 189.5 min in the HAT group and 62.17 min, 17 min, and 202 min in the non-HAT group, respectively (no significant differences: *p* = 0.78, *p* = 0.89, and *p* = 0.332, respectively).

Among 27 patients who developed HAT, 16 (59.3%) received a graft with one artery, and 11 (40.7%) received a graft with two or more arteries. In the non-HAT group, 263 grafts presented with one artery (70.5%), and 110 grafts (29.5%) presented with two or more arteries. In our material, double arterial anastomosis was performed in 36 patients. HAT developed in three of these children (8.3%), which is not significantly different from patients with single HA anastomosis (6.6%) (*p* = 0.724). The incidence of patients with a hepatic artery anastomosis diameter below 2 mm was significantly higher in the HAT group (*p* = 0.0026).

In one patient from the HAT group and four patients from the non-HAT group, two arterial stump reconstructions of the hepatic artery were performed during the back table preparation (end-to-side anastomosis of accessory HA to the main HA). In three patients, all from the non-HAT group, polytetrafluoroethylene (PTFE) prosthetic arterial conduits were used. In two cases, the arterial conduits were implanted into the infrarenal abdominal aorta. In one patient, the arterial conduit was anastomosed to the gastroduodenal artery and subsequently attached to the graft’s hepatic artery. Indications for the implantation of the arterial conduit were intimal dissection in the first patient, and the insufficient length of the hepatic artery in the second patient. In the third patient, according to the operative protocol, the diameter of graft artery was 3 mm, the patient artery was 1 mm in diameter, and we did not obtain measurable flow after reperfusion, so we decided to make a conduit to the infrarenal aorta with a PTFE graft. 

The occurrence of intraoperative HA flow dysfunction was significantly different between the groups (*p* = 0.0019). In the HAT group, arterial revision was performed in six patients (22.2%); reanastomosis was done in two, and the graft artery was washed with saline and heparin in three patients. In one case, we observed segmental ischemia, probably caused by a segmental artery missed during harvesting. In the non-HAT group, 16 children (4.3%) developed intraoperative HA flow dysfunction. In four patients, reanastomosis was performed; ten patients underwent HA revision with washing the graft artery with saline and heparin. In two cases, segmental liver ischemia was noted. 

Graft arterial flow was reestablished in five out of six patients from the HAT group and 14 out of 16 patients from the non-HAT group who underwent surgical revision during transplant surgery.

Anastomosis of the graft biliary duct to the Roux-en-Y loop was performed in 25 patients (92.6%) in the HAT group and 328 children (87.9%) in the non-HAT group.

The abdominal wound was closed primarily in 18 patients (66.7%) from the HAT group and in 191 (51.2%) from the non-HA group. Delayed abdominal closure was similar in both groups (*p* = 0.12).

### 3.2. Management of HAT

**Figure 2:**
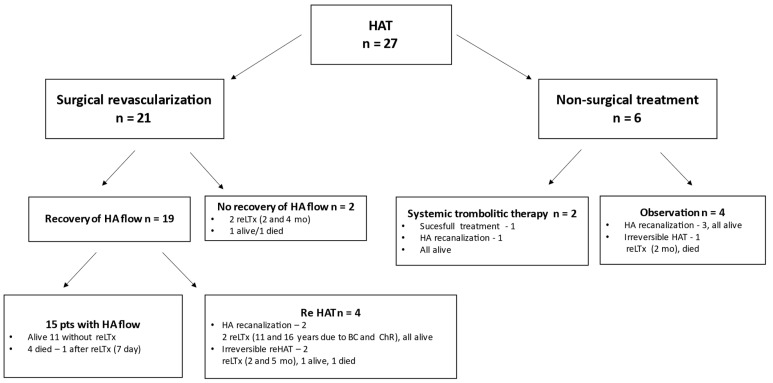
Management of HAT.

HAT management procedures are listed in Figure 2. 

Among 27 patients who developed HAT, 21 patients (77.8%) underwent urgent surgical revision. In five cases, arterial flow was restored after washing the graft artery with saline and heparin. In 16 children, thrombectomy was performed with additional r-TPA infusion in 11 of them. In five patients, reanastomosis was necessary. The arterial flow was not successfully restored in two children. Both of them required subsequent retransplantation two and four months after LDLT, respectively. One of them died due to multiple organ dysfunction syndrome (MODS). Recurrent HAT developed in four patients after successful surgical revascularization. Even though two of these children exhibited spontaneous intrahepatic arterial flow restoration, they still required a retransplantation 11 and 16 years after LDLT. Both of them remain alive. Two patients with irreversible recurrent HAT underwent liver retransplantation (two and five months after LDLT). One of them died from MODS. In 15 patients, long-term patency was achieved, and 11 of them are still alive. Four children died with a patent hepatic artery. Three children developed graft failure and died due to MODS (two patients) and sepsis (one patient). One with concomitant portal vein thrombosis underwent early retransplantation seven days after the first LDLT and died from MODS. 

The success rate of surgical revascularization was 61.9%. Thirteen patients out of the 21 who underwent surgical revascularization survived with the revascularized graft for more than 12 months. Two of them needed a second graft (six and 11 years after the first LDLT).

In six patients (22.2%) who developed HAT, surgical revision was not attempted. Four of them had eHAT. In one case, HA reconstruction was necessary at the back table. This patient had two HA stumps and end-to-side anastomosis of the accessory HA to the main HA was performed. This patient developed irreversible HAT six days after LDLT. Surgical revascularization was not performed after HAT because of concomitant sepsis. The patient underwent a liver retransplantation due to a liver abscess two months after LDLT, and subsequently died from MODS. In two patients, who developed HAT at one and 13 days after LDLT, the HA was already revised at LDLT surgery due to inadequate or absent flow, and surgical revision after HAT was impossible for technical reasons. In one patient, the HA was very short and small (artery diameter of 1 mm). In this patient, during LDLT, the immediate revision of the HA was performed with a Fogarty catheter. The second patient underwent a failed attempt to create a conduit due to the intimal dissection of the abdominal aorta at the time of LDLT. HA reanastomosis was performed and the gastroduodenal and splenic arteries were ligated to improve arterial inflow to the liver graft. The recanalization of the HA occurred 56 and four days after HAT diagnosis, respectively, in both patients.

One patient who developed HAT 13 days after LDLT was treated with an intravenous infusion of r-TPA at a dose of 0.01 to 0.05 mg/kg/h for 7 days. Spontaneous intrahepatic arterial flow recovery was detected 21 days after HAT diagnosis by Doppler ultrasound.

In the remaining two patients, late HAT occurred (38 and 116 days after LDLT), and their graft function was not impaired. One patient was treated with an intravenous systemic infusion of r-TPA at a dose of 0.01 to 0.05 mg/kg/h and the successful recanalization of the HA was observed after 7 days. In the second, spontaneous intrahepatic arterial flow recovery was detected 13 days after HAT diagnosis. 

We did not observe bleeding in patients treated with a systemic infusion of r-TPA. 

### 3.3. Associated Complications, Patient and Graft Survival

The incidence of biliary complications (biliary leaks and biliary stenosis) was 25.5% (n = 102). No difference in the occurrence of biliary leak from biliary anastomosis or the cut surface of the liver was found among the groups (Table 4). Eight patients (29.6%) from the HAT group and 30 children (8.0%) in the non-HAT group developed biliary stenosis. The rate of biliary stenosis was significantly higher in the HAT group (*p* = 0.0002). 

Retransplantation was performed in eight patients (29.6%) from the HAT group. In five of them, irreversible HAT was observed, and they underwent a second LT between two and five months after primary LDLT. The indications for liver retransplantation in those five patients were poor graft function in three patients, liver abscess in one case, and severe acute and subsequent chronic rejection in one patient. One child with successfully restored HA flow needed retransplantation due to concomitant portal vein thrombosis seven days after LDLT. Two patients, after the attempted surgical revascularization and delayed recanalization of HA, underwent liver retransplantation 11 and 16 years after LDLT due to biliary cirrhosis and chronic rejections, respectively. 

All patients who underwent a second LT received a graft from a deceased donor. Six patients received a whole liver, and two received grafts reduced to left lateral segments. Three of them required the use of an arterial conduit for HA reconstruction. 

A comparison of the numbers of retransplantations between the HAT and the non-HAT group during the entire follow-up period shows that the incidence of retransplantation was significantly higher in the HAT group (*p* < 0.0001).

**Figure 3:**
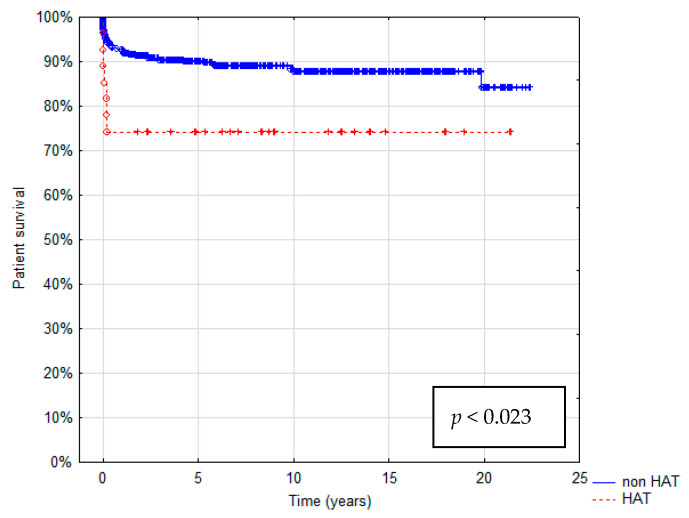
Kaplan–Meier patient survival curve.

**Figure 4:**
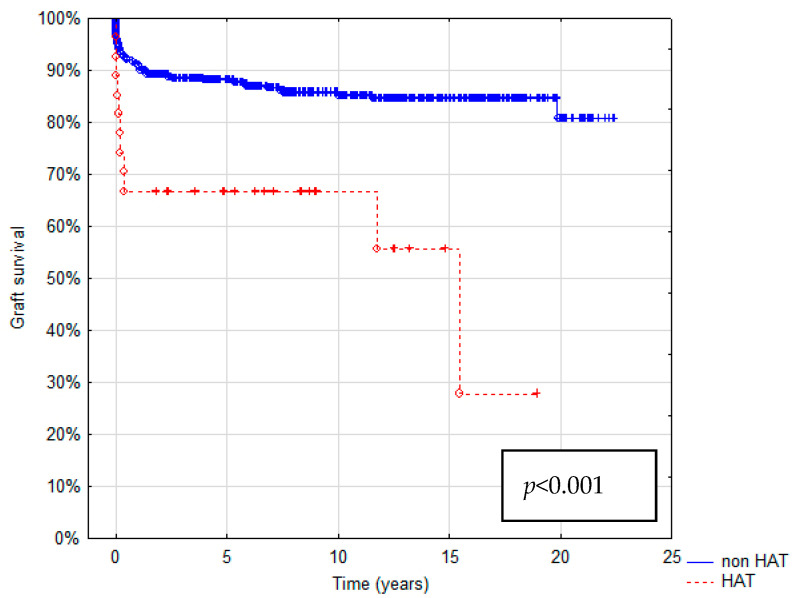
Kaplan–Meier graft survival curve.

Patient survival after 1, 5, and 10 years was 74.1%, 73.9%, and 61.5% in the HAT group and 93.3%, 89.2%, and 85.6% in the non-HAT group, respectively. In the HAT group, graft survival was 66.7%, 63.6%, and 46.2%. In the non-HAT group, it was 91.2%, 87.2%, and 81.7% at 1, 5, and 10 years, respectively. Estimated patient and graft survival are shown as Kaplan–Meier Curves in Figure 3 and Figure 4. The HAT group was characterized by the significantly worse survival of both patients and grafts (*p* < 0.023 and *p* < 0.001, respectively).

Six patients with HAT died due to MODS, four of whom had undergone liver retransplantation (Table 5). One patient died from infectious complications. In the non-HAT group, the major causes of death were infection (13 patients) and multiple organ dysfunction syndrome (MODS) (12 patients). Six children died from the recurrence of a liver tumor.

In summary, eight patients out of 27 with HAT underwent retransplantation. Twenty patients (74.1%) are alive, 16 of them (59.3%) having the original liver graft and four (14.8%) of them with a second graft. Seven patients who developed HAT (25.9%) died, including four children, after liver retransplantation.

## 4. Discussion

In the present analysis, we show that 6.75% of LDLT were complicated by HAT, which is similar to other studies. Based on multicenter data from the Society of Pediatric Liver Transplantation (SPLIT), Ebel et al. evaluated 3801 first-time pediatric liver transplant recipients [4]. In this group, 7.4% of patients developed HAT within the first 90 days of the transplantation. Bekker et al. reported a 4.4% incidence of HAT after liver transplantation during the first 30 days. When they compared the incidence of HAT between children and adults, they concluded that it was significantly higher in children (8.3%) than in adults (2.9%) [2]. 

Several surgical and nonsurgical risk factors of HAT have been described in the literature [5]. Surgical factors associated with HAT may include technical problems in creating anastomoses, performing complex back-table arterial reconstruction, and using an aortic conduit for arterial reconstruction and Roux-en-Y for biliary reconstruction [6]. Other technical causes that increase the risk of HAT include stenosis of the HA anastomosis and kinking or angulation of the HA. The actual cause of HAT is mostly unknown. One explanation may be the smaller diameter of the HA and technical difficulties during anastomosis. In our group, the number of patients with an anastomotic diameter below 2 mm and patients who developed intraoperative HAT was significantly higher in the HAT group. 

When the graft had more than one artery, we preferred reconstructing all of the HA. We consider making double arterial anastomoses with graft double arteries to be a very important protection against HAT, which may develop in one of these arteries. Seda-Neto et al. reported that performing two HA anastomoses during LDLT reduced the risk of HAT [1]. In our study, we did not observe the impact of multiple HA anastomoses on the incidence of HAT, similarly to other authors [7,8].

An arterial conduit is an effective rescue option in case of low arterial inflow, arterial wall dissection, or inadequate HA length. In the study by Bakker et al., the use of an arterial conduit is considered to be a risk factor for HAT [2]. Similar observations were reported by other authors [9,10]. In contrast, other authors have shown that the use of arterial conduits did not influence the incidence of HAT [11]. In our cohort, three patients had prosthetic arterial conduits (PTFE); none of them developed HAT after LDLT. We used PTFE conduits because during these LDLT, we did not have access to an arterial graft from the living donor or cryopreserved arterial grafts from a deceased donor. 

Similar to our results, Seda-Neto et al. reported an increased risk of HAT in patients who presented with an intraoperative HAT [1]. This may be caused by technical problems during arterial anastomosis (anastomotic kinking or angulation, anastomosis under tension), arterial spasm, or intimal dissection. To prevent the intraoperative occurrence of HAT, in all of our patients, we routinely perform an ultrasound examination.

Kutluturk et al. reported that biliary anastomosis to Roux-en-Y loop during LDLT was a risk factor for the development of eHAT [7]. They hypothesize that this could be caused by the compression of the HA anastomosis by the Roux-en-Y loop. Similar results were shown by Silva et al. [10]. In our material, biliary anastomosis to Roux-en-Y loop was performed in patients with biliary atresia and in recipients in whom the use of a native bile duct was not feasible (e.g., too short), or when there were multiple bile ducts in the graft. To date, we have not confirmed any effect of the type of biliary reconstruction on the incidence of HAT. However, we strongly believe it is crucial to avoid stretching or kinking the HA during maneuvers used to expose the graft during biliary anastomosis.

An important factor affecting the incidence of HAT is graft size. In patients with large grafts, the risk of vascular complications including HAT is higher [12,13]. In such situations, graft compression and compartment syndrome may develop, leading to the worsening of arterial flow with subsequent HAT. Another study indicated that a GRWR of 1.1% or less can be an independent risk factor for HAT [1]. We did not observe a negative impact of recipient body weight or GRWR on the incidence of HAT in our material. Our previous analysis demonstrated that in patients with a GRWR > 5%, the possibility of graft loss and recipient mortality is higher [14]. In this study, GRWR > 5 % was an independent risk factor for death in patients after LDLT, most probably due to the multifactorial effect of inadequate graft perfusion and graft dysfunction, the prolonged duration of mechanical ventilation and intensive care unit stay, and infectious complications. To prevent vascular complications in patients with higher GRWR, we left the abdomen open. Another strategy to solve the problem of graft mismatch, used in 13 patients from our group, was the reduction of liver grafts into monosegments, decreasing GRWR and reshaping grafts that were too thick.

In our previous study, we successfully showed that LDLT is a lifesaving procedure for pediatric patients with acute liver failure [15]. In the above research, we found that acute liver failure (ALF) was a more common indication for LT among patients with HAT. This might have been related to the smaller diameter of HA in patients with acute liver failure compared with relatively overdeveloped arteries in patients with chronic liver failure. Patients with ALF received more transfusions of blood products perioperatively due to coagulopathy, which was also described as a risk factor for HAT development [10]. These observations oppose other studies, which showed that children transplanted for acute liver failure were less likely to develop HAT than children with biliary atresia [4].

In the study of Bekker et al., the median time to HAT detection was 6.9 days (ranging from 1 to 17.5 days postoperatively) and was similar to our results [2]. These authors also found a correlation between the early occurrence of HAT and a high success rate of revascularization attempts whenever a Doppler ultrasound was performed daily. Others have also reported a significant decrease in the incidence of biliary complications and graft loss after the early detection of HAT in Doppler ultrasound [16].

There are three treatment strategies for HAT: revascularization, retransplantation, and observation. In the current study, surgical revascularization for HAT was attempted in 77.8 % of patients, with a success rate of 61.9%. In a study by Warnaar et al., 32 patients (13.7%) developed HAT [17]. In 16 children (50%), immediate surgical thrombectomy was attempted with a graft salvage rate of 38%. Bekker et al., in cases of early HAT after liver transplantation, reported that revascularization was attempted in 54.1% of the children with a success rate of 55.6% [2]. In our series, surgical revision was performed in a higher number of patients, with a satisfying success rate. During the surgical intervention, the HA should be carefully inspected for thrombus. In the case of HAT, a prompt surgical thrombectomy through a branch of the recipient’s HA or opening of the HA anastomosis should be performed. Surgical factors, such as stenosis, kinking, or angulation, should be corrected during the redo of HA anastomosis. 

Two of our patients underwent successful revascularization with systemic thrombolytic therapy with an intravenous systemic infusion of r-TPA. Both of them are alive and maintain the original graft. The number of patients who successfully received this treatment is very limited in the literature. Feirer et al. reported two pediatric patients with failed surgical revascularization who were subsequently treated with systemic r-TPA infusion (0.3 mg/kg/h for 6 hours for five days) [18]. In both patients, the recanalization of HA was observed, and they did not need a second transplantation. 

Bekker et al. described 24 pediatric patients from 10 different studies who developed HAT and did not receive any treatment for it [2]. The overall mortality in this group was 58.3%. In our study, only four such patients with HAT were observed. Three of them had spontaneous recanalization and are still alive, maintaining their original grafts. 

Two factors are crucial for HAT development and the patients’ final outcome. The first is the successful and immediate restoration of arterial flow. The second factor is the time of HAT occurrence. Rabkin et al. reported that after eHAT in a liver allograft without an arterial inflow from collaterals, arterial ischemia may cause the necrosis of the transplanted liver and the development of biliary leaks or stricture, leading to graft failure [19]. Stringer et al. showed that arterial collaterals may appear after three weeks post-surgery and developed earlier in the presence of a cut surface of the liver or biliary anastomosis to the Roux-en-Y loop [20]. They also concluded that arterial supply from collaterals may salvage the transplanted liver. In another study, Gu et al. detected arterial inflow from collaterals two weeks after early HAT, but the blood flow was significantly lower than after successful revascularization (low resistive index and low peak systolic velocity) [21]. 

The incidence of biliary complications after HAT is higher due to the impairment of vascularization of the bile duct derived from HA. Darius et al. reported 429 primary pediatric liver transplantations, including 203 LDLT [22]. They observed biliary complications in 98 patients (23%), including 47 strictures (78%) and 13 fistulas (22%) from biliary anastomosis. According to their study, HAT increased the risk of biliary complications. In our material, the overall incidence of biliary complications was similar. Similarly, more patients who developed HAT presented biliary strictures in the transplanted liver.

Liver retransplantation used to be the main approach for HAT treatment in the early era of liver transplantation. Tzakis et al., reported on a cohort of 309 patients after liver transplantation [23]. HAT occurred in 22 patients (7.1%). Mortality after liver retransplantation was 27.3% and 72.7% without retransplantation. The poor availability of pediatric liver donors limits the number of necessary retransplantations. That is how the revascularization of the HA became the first treatment option providing equally good results. Ackermann et al. reported that successful surgical revascularization significantly improved graft survival. They reported a 20-year graft survival as high as 77% compared with 24% for grafts when revascularization was not performed or failed [24]. 

HAT remains one of the main indications for liver retransplantation. Ebel et al. reported a cohort of 3801 pediatric patients undergoing their first liver transplantation [4]. HAT developed in 281 patients (7.4%) within 90 days after transplantation, and 121 of them needed retransplantation. They also observed a high incidence of HAT after the second liver transplantation (20.7%). In a study by Bordeaux et al., HAT was a primary indication for early retransplantation in 33% of patients. Only 16% of patients who underwent late liver retransplantation presented with HAT [3]. They showed that both graft and patient survival rates after the second liver transplantation were lower when compared with children undergoing primary liver transplantation.

In our study, the most common indications for liver retransplantation after HAT were complications following failed revascularization. Only one patient underwent liver retransplantation within 30 days after the first transplantation due to concomitant portal vein thrombosis. Only four of our patients after liver retransplantation are alive, and these results are similar to those reported by other authors [25].

Hepatic artery thrombosis after LDLT is a very serious and life-threatening complication. The technical problems with creating an anastomosis on 1.5 to 2.5 mm arteries, sometimes multiple, are well recognized by pediatric transplant surgeons. In our opinion, sharing our experience, particularly achieved on a relatively large group of transplants, with detailed descriptions of procedures and protocols, and results achieved by our team will hopefully add to the discussion on best practices in LDLT. Careful surveillance with repeated ultrasound examination allowed us to avoid HAT development in patients with significant deterioration of intrahepatic arterial flow.

## 5. Conclusions

The close monitoring of hepatic artery flow with Doppler ultrasound during the critical period of two to three weeks after LDLT and the immediate intervention in the form of surgical revascularization may alleviate the increased risk of biliary stenosis, graft loss, and the need for retransplantation resulting from HAT.

## Figures and Tables

**Table 1:** Underlying liver disease of patients.

Diagnosis	HAT (n = 27)	Non-HAT (n = 373)	*p*-Value
Cholestatic disease	16 (59.3%)	248 (66.5%)	*p* = 0.84
Biliary atresia	14 (51.9%)	222 (59.5%)	*p* = 0.43
Liver tumors	2 (7.4%)	47 (12.6%)	*p* = 0.43
Acute liver failure	5 (18.5%)	28 (7.5%)	*p* < 0.05
Metabolic disorders	1 (3.7%)	11 (2.9%)	*p* = 0.82
Others	3 (11.1%)	39 (10.5%)	*p* = 0.91

**Table 2:** Characteristics of recipients and donors.

Characteristics	HAT (n = 27)	Non-HAT (n = 373)	*p*-Value
Recipients			
Female gender (%)	16 (59.3%)	197 (52.8%)	*p* = 0.28
Age (years); median (range) <1 year	1.3 years (5 months–10 years)	1.2 years (13 days–15 years)	*p* = 0.68
	11 (40.7%)	156 (41.8%)	*p* = 0.91
Body weight (kg); median (range)	9.5 (5.6–26)	9.5 (3.1–47)	*p* = 0.52
PELD score; median (range)	12.5 (−9–43)	14 (−11–49)	*p* = 0.83
AB0-incompatibile LDLT	4 (14.8%)	58 (15.5%)	*p* = 0.92
Urgent LDLT	5 (18.5%)	39 (10.5%)	*p* = 0.2
Donors			
Female gender (%)	16 (59.3%)	252 (67.6%)	*p* = 0.38
Age (y); median (range)	32 (22–44)	31 (5–57)	*p* = 0.81
Body weight (kg); median (range)	68 (43–88)	65 (19–110)	*p* = 0.55

**Table 3:** Surgical variables.

Operation	HAT (n = 27)	non-HAT (n = 373)	*p*-Value
Graft type			
Monosegment	0	13	*p* = 0.32
Left lateral segment (segment II + III)	24	321	*p* = 0.68
Left lobe (segment II + III + IV)	3	38	*p* = 0.88
Right lobe (segment V + VI + VII + VIII)	0	1	*p* = 0.79
Graft weight (g); median (range)	256 (160–436)	268 (131–919)	*p* = 0.78
GRWR (%); median (range)	2.2% (1–4.9)	2.7% (0.9–10.6)	*p* = 0.19
Biliary anastomosis			
Roux-en-Y hepaticojejunostomy	25 (92.6%)	328 (87.9%)	*p* = 0.58
Duct to duct anastomosis	2	45	*p* = 0.58
Vascular anastomosis time (min); median (range)	60.5 (49–100)	62 (40–152)	*p* = 0.78
HA anastomosis time (min); median (range)	17 (9–31)	17 (5–70)	*p* = 0.89
Cold ischemic time (min); median (range)	189.5 (65–397)	202 (85–431)	*p* = 0.332
Number of HA in graft			
One HA (%)	16 (59.3%)	263 (70.5%)	*p* = 0.19
Two or more HA (%)	11 (40.7%)	110 (29.5%)	*p* = 0.19
Two arterial anastomoses (%)	3 (11.1%)	33 (8.8%)	*p* = 0.69
Diameter of HA anastomosis < 2 mm (%)	6 (22.2%)	24 (6,4%)	*p* = 0.0026
Back table HA reconstruction (%)	1 (3.7%)	4 (1.1%)	*p* = 0.23
Arterial conduit (%)	0	3 (0.8%)	*p* = 0.64
Intraoperative HA revision after reperfusion	6 (22.2%)	16 (4.3%)	*p* = 0.0019
Delayed abdominal wall closure *	18 (66.7%)	191 (51.2%)	*p* = 0.12

* Patients who received a second transplantation or died during hospital stay after LDLT are excluded.

**Table 4:** Biliary complications and retransplantation following LDLT (n,%).

	HAT (n = 27)	Non-HAT (n = 373)	*p*-Value
Biliary leaks			
Biliary anastomosis	3 (11.1%)	42 (11.2%)	*p* = 0.98
Cut surface of the liver	3 (11.1%)	16 (4.3%)	*p* = 0.11
Biliary stenosis	8 (29.6%)	30 (8.0%)	*p* = 0.0002
Retransplantation	8 (29.6%)	13 (3.5%)	*p* < 0.0001

**Table 5:** Causes of patient death.

Cause of Death	HAT (n = 27)	Non-HAT (n = 373)
Infections	1	13
Multiple organ dysfunction syndrome	6	12
Malignancy/tumor recurrence	0	6
Acute rejection	0	1
Graft versus host disease	0	1
Central nervous system complications	0	6
Gastrointestinal bleeding	0	1
Allograft dysfunction	0	1
Autoimmune thrombocytopenia	0	1
Non-medical	0	1
Total (n,%)	7 (25.9%)	43 (11.5%)

## Data Availability

Most of the relevant data are contained in the paper. Most of the output data were taken from the Polish National Transplant Registry at https://rejestrytx.gov.pl/tx/ (accessed on 1 April 2022). Since the data collected in the Registry is sensitive and, thus, protected by law (the Personal Data Protection and the Medical Records Act), access to the database is limited; it can be accessed only upon meeting registration criteria. The Registry is under the supervision of the Polish Transplant Coordinating Centre “Poltransplant”, funded through the budget of the Polish Ministry of Health. Since the registry is only available to a limited number of healthcare professionals, working in transplantation units across Poland, access to the database is impossible for people not involved directly in transplantation and the coordination process on the national level. All of the patients’ medical histories and other vital information used in the creation of the database are available directly at the Children’s Memorial Health Institute, Warsaw, Poland, after contact with Piotr Socha at P.Socha@ipczd.pl.
